# Loot

**Published:** 1872-07

**Authors:** 


### Loot.

***Clinical Thermometry.***

Dr. Lucius D. Bulkley has presented a lengthy paper on this subject to the New York Medical Society. It is based on a prize essay he has obtained. The conclusions of the author, based on 337 cases of which a record is kept, not only of the temperature but of the pulse and respiration, are as follows:

1. The body heat is maintained in health, under all conditions, at the uniform standard of 98.4° Fahr.
2. Any constant deviation from this constitutes disease.
3. A return to and continuance at this standard marks the termination of the disease.
4. A single high temperature is important.
5. The changes of temperature in diseases follow definite and known courses.
6. Variations from these typical ranges of temperature in disease are significant, as indicating a disturbing cause.
7. An irregular course is more unfavorable than a uniformly high range of temperature.
8. Different temperatures characterize different diseases, and various days of the same disease.

9. Although a high temperature indicates a more severe attack, no heat under 109° can be considered surely fatal.

10. The daily study of the pulse and respiration in connection with the temperature is of great assistance.

11. When the temperature and general symptoms agree, but the pulse disagrees, the two former are to be relied on.

12. When the pulse and general symptoms agree in indicating unfavorably, the temperature cannot be relied on, if contradictory, unless the improvement in respect to temperature is marked and persistent.

13. When the pulse and general symptoms agree in a favorable indication, a high or rising temperature should arrest attention.

14. All other means of investigation should be used in connection with the temperature to obtain the greatest benefit from the latter.

15. The continuous daily record of the three vital signs here represented, in the way exhibited, affords much aid in diagnosis, prognosis, and treatment of disease, by the presentation to the eye of its history in these respects.

16. The systematic record of these three points may assist in determining, at some future day, the vexed question whether the type of disease is changing, by preserving pictures which can be easily compared.

### *Treatment of Pruritus Vulvæ.*

Those who have had any experience in the treatment of this troublesome affection will learn with interest that Mr. McGrath states (*Canada Lancet*, November, 1871,) that he has found the following, applied by means of a soft sponge after ablution, morning and evening, attended with the most satisfactory and speedy result:

| | | |
|---|---|---|
| R. | Biborate of Soda, - - - - | gr. ij. |
| | Hydrochlorate of Morphia, - - | gr. xx. |
| | Hydrocyanic Acid, - - - - | gr. j. |
| | Glycerine, - - - - - | f oz. j. |
| | Distilled rose-water, - - - - | f oz. viij. |

*Nashville Jour. of Med. and Surg.*

### *Chinese Surgery.*

John G. Keer, M.D., Chief Surgeon of the Canton Hospital, writes that the department of surgery in this empire can scarcely be said to exist, beyond the application of caustics and plasters to tumors and ulcers, and of poultices to broken bones. They are entirely helpless. There is no native doctor in all China who can give aid in case of accident or disease which requires manual or instrumental interference. The inestimable benefits of operative surgery, in all its branches, are unknown to them. They are entirely without surgical instruments and all the apparatus which modern ingenuity has applied to the relief of injuries, deformities and disease.—*Detroit Review of Medicine and Surgery.*

### *Gelseminum in the Treatment of Irritable Bladder.*

Dr. W. Scott Hill, Augusta, Maine, (Am. Jour. Med. Sciences, January, 1872), gives five cases of "irritable bladder" successfully treated with gelseminum. In all the cases the same symptoms were present, namely: frequent calls to void the urine, which was small in quantity, often passed *guttatim*, and excessive pain attending micturition. He used Tilden's Fluid Extract of Gelseminum. His usual prescription was:

R. Potassi Bromid., - - - - gr. iv.
Potass. Carbon., - - - - gr. iij.
Fluid Ext. Gelsem., - - - - m. x.
Aquæ., - - - - - oz. ij. M.
S. This quantity every 4 or 6 hours.

—*Georgia Med. Companion.*

### *The "American Plan"*

Is unquestionably best for America. Electing judges by the people, every one knew would ruin the country, but it didn't. A thousand things contrary to reason, and undoubtedly wrong in the abstract, in America naturally rise up and become the heads of as many corners. The idea of the government of the country was a wrong idea, and was demonstrated to be so by the best talent abroad and at home; but it was American. Amid the clash and clatter, all over America, for the last quarter of a century, upon reform in medical teaching, our feeble voice has, all along the line, proclaimed, "probably medical teaching in America is all wrong, but it has been developed by an American necessity, and is eminently American, and therefore, beyond all doubt, the best for America, and the tendency is infinitely greater for it to become more so than less so. The sessions are too long, the number of teachers and number of lectures too great. We want more demonstration and less gas. The gas element in all of our colleges is six times as long as the demonstration element. The gas is a remnant of foreign barbarism, and is not at all American, and is bound yet to escape through an American pin-hole."

Harvard lost one hundred and five students last session, by what exchanges call "the salutary reform," that is, three sessions. Three sessions, and Greek and Latin, look well, but they are not American. Our exchanges think Harvard will continue this "salutary reform," though it reduces her classes to zero; but we know better—Harvard is not going to do any such thing, but Harvard will return to her old way. Do we not all know that the University of Pennsylvania got on stilts once, and lengthened her session to six months, her rival poling along her old American keel at four months? Never mind, said the friends of the former, looking upon her empty benches, she will hold out to the last. Not a bit of it, we suggested; she will do a more sensible thing—she will get off her stilts and forswear any such unnatural method of locomtion for life; and she did.

When we consider the number and the value of medical books, (which, notwithstanding, are not to be forced upon students at college, as text-books, when, of all seasons, they have least time to read them, and of all times they can least afford money to buy them); we say, considering the number and value of medical books now, compared with what medical libraries exhibited forty years ago, with opportunity, out of college, afforded by hospitals and clinics, we are amazed that medical teachers still ingeniously contrive successful means to keep students away from them for months, only to hear, from hard benches, these gentlemen gas by the hour.

The great trouble of innovators is, that they will not comprehend that medical abilities are *never* developed by medical colleges. If a man becomes a great physician, he makes himself so after he quits college, and independently of anything he was taught there.

A teacher with his belly full of fire, and every nervous fibril in his organism astrut with electricity, with a memory faithful as a handmaid to his genius, and to the threshold of whose storehouse of learning the writers of all countries and all ages have lain down their contributions; such a man, in an active state of eruption, with lightnings flashing about his mouth, and lava, at a white heat, pouring over his beard, and scintillating among an audience, who, though spell-bound, have each a half-dozen able-bodied *Amens* struggling in his elongated throat, and, like a lighted shell, ready to explode; yes, such a man may inspire one to struggle on amid poverty, neglect, and contumely, till the day-star of promise shall peep over his horizon, and beckon him to triumph and glory; but it is the one man and not the college, to whom he will ascribe his regeneration.

The ways of America are best for America, and while they will naturally adapt themselves to the necessities of each age, they will neither be rough-hewed nor shaped by any one, being, indeed, the logic of events, and therefore inexorable.—*Nashville Journal.*

